# The effect of dietary supplementation with *Nigella sativa* (black seeds) mediates immunological function in male Wistar rats

**DOI:** 10.1038/s41598-021-86721-1

**Published:** 2021-04-06

**Authors:** Hany Salah Mahmoud, Amani A. Almallah, Heba Nageh Gad EL-Hak, Tahany Saleh Aldayel, Heba M. A. Abdelrazek, Howayda E. Khaled

**Affiliations:** 1 Center of Scientific Foundation for Experimental Studies and Research, Ismailia, 41511 Egypt; 2 Anatomy and Embryology Department, Faculty of Medicine, Suez Canal University, Ismailia, 41522 Egypt; 3 Zoology Department, Faculty of Sciences, Suez Canal University, Ismailia, 41522 Egypt; 4 Nutrition and Food Science, Department of Physical Sport Sciences, Princess Nourah bint Abdulrahman University, Riyadh, 11671 Saudi Arabia; 5 Department of Physiology, Faculty of Veterinary Medicine, Suez Canal University, Ismailia, 41522 Egypt; 6 Zoology Department, Faculty of Sciences, Suez University, Ismailia, 43533 Egypt

**Keywords:** Biochemistry, Cell biology, Immunology, Physiology, Plant sciences, Zoology, Biomarkers, Diseases, Health care, Medical research

## Abstract

This experiment aimed to investigate the effect of dietary *Nigella sativa* on the cell-mediated immune response. Eighteen male Wistar rats were divided equally into a control group and treated groups that received black seeds at rates of 30 and 50 g/kg in the diet (Sa30 and Sa50 groups, respectively, for 30 days. The weight gain, feed intake, feed conversion ratio (FCR), and cell-mediated immune response were monitored after the injection of 0.1 mL of 10% phytohemagglutinin (PHA). The intumesce index, serum total antioxidant capacity (TAC), catalase (CAT), interleukin-12 (IL-12), gamma interferon (γ-IF) and tumor necrosis factor alpha (TNF-α) were determined. Histopathological examination and an immunohistochemistry analysis of splenic caspase-3 and CD8 were performed. *Nigella sativa* significantly improved the weight gain and FCR. Intumesce index of Sa50 group was significantly increased. *Nigella sativa* significantly increased TAC, CAT, IL-12, γ-IF and TNF-α. A histological examination of PHA-stimulated foot pads showed increased leukocyte infiltration and edema in a dose-dependent pattern. Splenic caspase-3 and CD8 showed significant decreases and increases, respectively, in the Sa30 and Sa50 groups. The results indicate that *Nigella sativa* seeds exhibit immunostimulatory function through their antioxidant potential, induction of cytokine production, promotion of CD8 expression and reduction of splenic apoptosis.

## Introduction

Immunostimulants enhance the cell-mediated immune response by activating antigen-specific cytotoxic T lymphocytes and phagocytes and discharging several cytokines toward antigens to achieve a therapeutic response^1^. Immunostimulants overwhelm the immunosuppressive effects of infectious agents and either induce stress on the interface and/or damage the function of immune cells^2^. Diverse substances, including plants and animal derivatives, microbial products, hormones, synthetic chemicals and vitamins, exert immunostimulatory effects. Herbs, plant extracts and animal-originated products are widely used because they are not expensive, can be easily obtained and act against a wide-ranging spectrum of pathogens^3^. The oral administration of plant or herbal extracts as immunostimulants is considered the best method for immunostimulation^4^.

Herbal medicines extracted from plants or plant extracts have historically been used to enhance health. In recent years, scientists have attempted to recognize the key ingredients of herbal medicines and to comprehend their mechanisms of action^5^. One of these herbal medicines is black seed, or *Nigella sativa* belonging to the family Ranunculaceae, which has a rich religious and historical background. This species has been grown and used in various parts of the world as a food additive, spice and remedy for a large variety of diseases, such as headaches, bronchial asthma, nasal congestion, toothache, allergies, back pain, hypertension, obesity, gastrointestinal troubles and numerous types of cancer. Additionally, *Nigella sativa* seeds can minimize fatigue and depression and increase the strength of the body. Moreover, due to its documented components, *Nigella sativa* exerts immunostimulatory effects in various inflammatory and immunologic diseases, such as experimental allergic encephalomyelitis, colitis, and arthritis^6^, and in sensitized animals, patients who suffer from asthma and victims of chemical warfare^7^.

Ahmed and El-Sayed^8^ indicated that dietary black seed supplementation successfully improves the body gain percentage, feed intake, and serum biochemical and immunological parameters in rats. *Nigella sativa* has numerous beneficial properties, including its ability to promote antioxidants^9^. Recent clinical and experimental studies have demonstrated that *Nigella sativa* extracts exert many therapeutic effects, including antidiabetic effects^10^, anti-inflammatory, anti-arthritic and anti-nociceptive activities in arthritic rats^11^. In addition, modern toxicological studies have shown that crude seed extracts and some of their active ingredients (volatile oil and thymoquinone) might exert protective effects against hepatotoxicity and nephrotoxicity caused by either chemical substances or diseases^12^. Moreover, *Nigella sativa* activates bone marrow and immune cells and increases the production of interferon, which results in defending normal cells against cell death by virus killing, destroying tumor cells and increasing the amount of antibody-generating B cells^13^.

The current study aimed to explore the effect of dietary *Nigella sativa* seed supplementation on growth performance parameters, the FCR, and the intumesce index as an indicator of immune function in Wistar rats. This aim was achieved by investigating the levels of total antioxidant and inflammatory cytokines, such as the total antioxidant capacity (TAC), catalase activity, interferon-gamma (IF-γ), interleukin-12 (IL-12) and tumor necrosis factor-alpha (TNF-α), in the rat model. Additionally, the histopathologic changes in the popliteal lymph nodes and spleen and the immunohistochemical expression of caspase-3 and CD8 in the spleen were examined.

## Results

### Analysis of phytochemical constituents of *Nigella sativa* seeds

The standard laboratory procedures for phytochemical screening demonstrated the presence of phenolic compounds (p-hydroxybenzoic acid, catechin, chlorogenic acid, ferulic acid, sinapic acid, p-coumaric, and kaempferol) (Table 1). The phenolic compounds found at the highest and lowest levels were p-hydroxybenzoic (69.685 μg/g) and kaempferol (1.277 μg/g), respectively. As shown in Table 1, the total phenol and flavonoid compounds and the 2,2‐diphenyl‐1‐picrylhydrazyl (DPPH) scavenging activities of *Nigella sativa* seeds equaled 2.077 mg gallic acid equivalents, 0.565 mg catechin equivalents and 1.367 g Trolox equivalents, respectively.

**Table 1:** Phenolic profile (μg/g), total phenols (mg GAE/g), Total flavonoids (mg CE/g) and antioxidant activity DPPH (mg TE/g) of black seeds *(Nigella sativa).*

Compound	Contents (μg/g)
*p*-Hydroxybenzoic acid (μg/g)	69.685
Catechin (μg/g)	32.824
Chlorogenic acid (μg/g)	2.554
Ferulic acid (μg/g)	6.628
Sinapic acid (μg/g)	21.979
*p*-Coumaric (μg/g)	45.147
Kaempferol (μg/g)	1.277
Total phenols (mg GAE/g)	2.077
Total flavonoids (mg CE/g)	0.565
DPPH (mg TE/g)	1.367

*GAE* gallic acid equivalents, *CE* catechin equivalents, *DPPH* 2‐diphenyl‐1‐picrylhydrazyl, *TE* Trolox equivalents.

The total phenol content is presented as gallic acid equivalents (GAE). The total flavonoid content is expressed as catechin equivalents (CE). The 2,2‐diphenyl‐1‐picrylhydrazyl (DPPH) scavenging activities of black seeds are presented as Trolox equivalents (TE).

### Growth performance parameters

No mortality or change in behavior was detected during the study. *Nigella sativa* positively affected the growth performance of rats. The rats given 30 and 50 mg/kg dietary *Nigella sativa* showed significantly (*P* < 0.05) increased weight gain compared with the control rats. The analysis of the data revealed that the FCR was significantly affected by *Nigella sativa*. The rats that received *Nigella sativa* at doses of 30 and 50 mg/kg basal diet showed significantly (*P* < 0.05) reduced FCR values compared with that of the control group (Table 2).

**Table 2:** Growth performance of rats, relative spleen weight and intumesce index of the groups fed *Nigella sativa* seeds (30 and 50 g/kg) and the control group.

Treatment groups	Weight gain (g)	Average food intake per week (g)	Feed conversion ratio (FCR)	Relative spleen weight (%)	Intumesce index
Control	23.00 ± 4.3^b^	367.50 ± 34.5	17.39 ± 1.9^b^	0.46 ± 0.02^b^	0.02 ± 0.01^b^
Sa30	37.10 ± 3.9^a^	390.81 ± 32.4	10.86 ± 1.9^a^	0.49 ± 0.01^b^	0.04 ± 0.02^ab^
Sa50	39.00 ± 2.5^a^	460.60 ± 26.0	12.67 ± 1.1^a^	0.60 ± 0.03^a^	0.06 ± 0.01^a^

The data are presented as the means ± SEs, n = 6 per group. Means within the same column with different superscript letters are significantly (*P* < 0.05) different according to one-way ANOVA followed by Duncan’s post hoc analysis*.* Sa30: group fed *Nigella sativa* at 30 g/kg diet; Sa50: group fed *Nigella sativa* at 30 g/kg diet.

### Relative spleen weight

Observations of the external appearance of the spleen yielded no noteworthy findings. The relative spleen weight of the rats belonging to the Sa50 group (*P* < 0.05) was higher than that of the rats in the control group. No difference in the relative spleen weight was found between the Sa30 group and the control group (Table 2).

### Intumesce index

A significant (*P* < 0.05) increase in the intumesce index of the ankle was found in the rats fed 50 g/kg *Nigella sativa* for 30 days compared with the control rats. However, the intumesce index of the Sa30 was not significantly different from those of the control and Sa50 groups (Table 2).

### Catalase and TAC

The serum catalase activity was significantly (*P* < 0.05) increased in the Sa30 and Sa50 groups compared with the control group. Additionally, the serum TAC was significantly (*P* < 0.05) increased in the Sa30 and Sa50 groups compared with the control (Table 3).

**Table 3:** Interferon-gamma (IF-γ), interleukin-12 (IL-12), tumor necrosis factor-alpha (TNF-α), and total antioxidant capacity (TAC) levels and catalase activity of the groups fed *Nigella sativa* seeds (30 g/kg and 50 g/kg) and the control group.

Treatment	Cytokine parameters			Antioxidant parameters	
	IF-γ (pg/mL)	IL-12 (pg/mL)	TNF-α (pg/mL)	TAC (mmol/l)	CAT (mmol/l)
Control	461.8 ± 3.9^c^	12.89 ± 0.1^c^	6.83 ± 0.03^c^	0.864 ± 0.003^c^	3.85 ± 0.01^c^
Sa30	566.5 ± 6.2^b^	15.72 ± 0.1^b^	8.804 ± 0.1^b^	1.070 ± 0.03^b^	4.27 ± 0.03^b^
Sa50	627.5 ± 1.9^a^	20.89 ± 0.1^a^	11.17 ± 0.1^a^	1.31 ± 0.02^a^	4.87 ± 0.01^a^

The data are presented as the means ± SEs, n = 6 per group. Means within the same column with different superscript letters are significantly (*P* < 0.05) different according to one-way ANOVA followed by Duncan’s post hoc analysis*.* Sa30: group fed *Nigella sativa* at 30 g/kg diet; Sa50: group fed *Nigella sativa* at 30 g/kg diet.

### IF-γ, IL-12 and TNF-α levels

The serum levels of IF-γ, IL-12 and TNF-α in the Sa30 and Sa50 groups were significantly (*P* < 0.05) higher than those in the control group (Table 3).

### Spleen histopathology and histomorphometry

**Figure 1:**
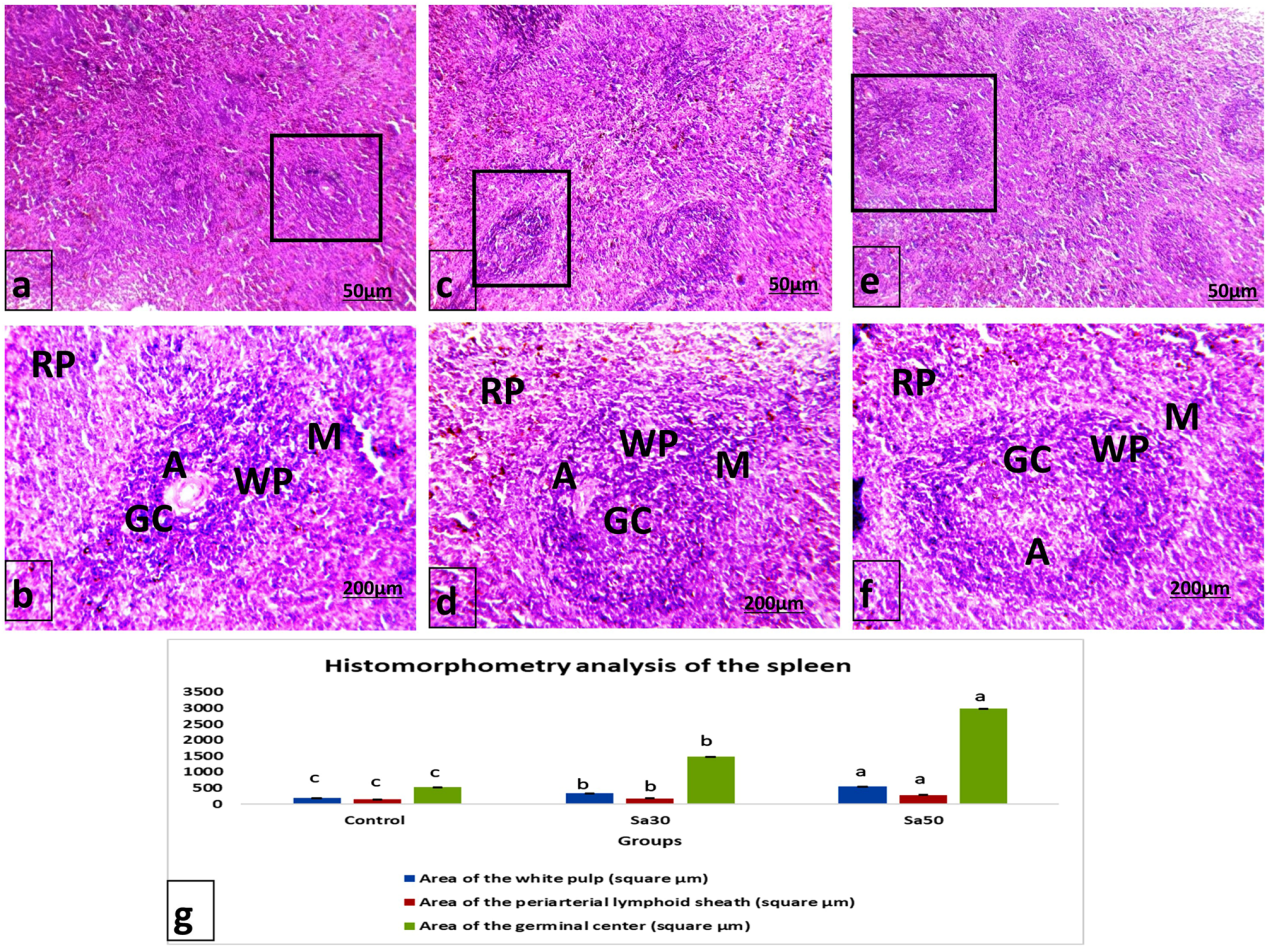
Photomicrograph of (**a** & **b**) the spleen from a control rat, (**c&d**) the spleen of a rat in the Sa30 group, and (**e** & **f**) the spleen from a rat in the Sa50 group showing marked histopathological changes in the spleen architecture with a distinct red pulp (RP), white pulp (WP) with a central arteriole (A) and a germinal center (GC) surrounded by marginal zone (M). H&E stain, ×200 magnification. (**g**) Histomorphometry analysis of the spleens of rats treated with *Nigella sativa* seeds. Different superscript letters represent significant differences at *P* < 0.05.

Microscopic examination of the control and *Nigella sativa*-treated spleens revealed no histopathological lesions in normal white and red pulp separated by marginal zones. The white pulp consists of a follicle with a pale germinal center and peripherally located central arterioles (Fig. 1). The histomorphometric analysis of the spleens showed that the areas of the white pulp, periarterial lymphoid sheath and germinal center in the spleens of the Sa30 and Sa50 groups were significantly increased compared with those of the spleen of control rats (Fig. 1), and this effect appeared to be dose-dependent.

**Figure 2:**
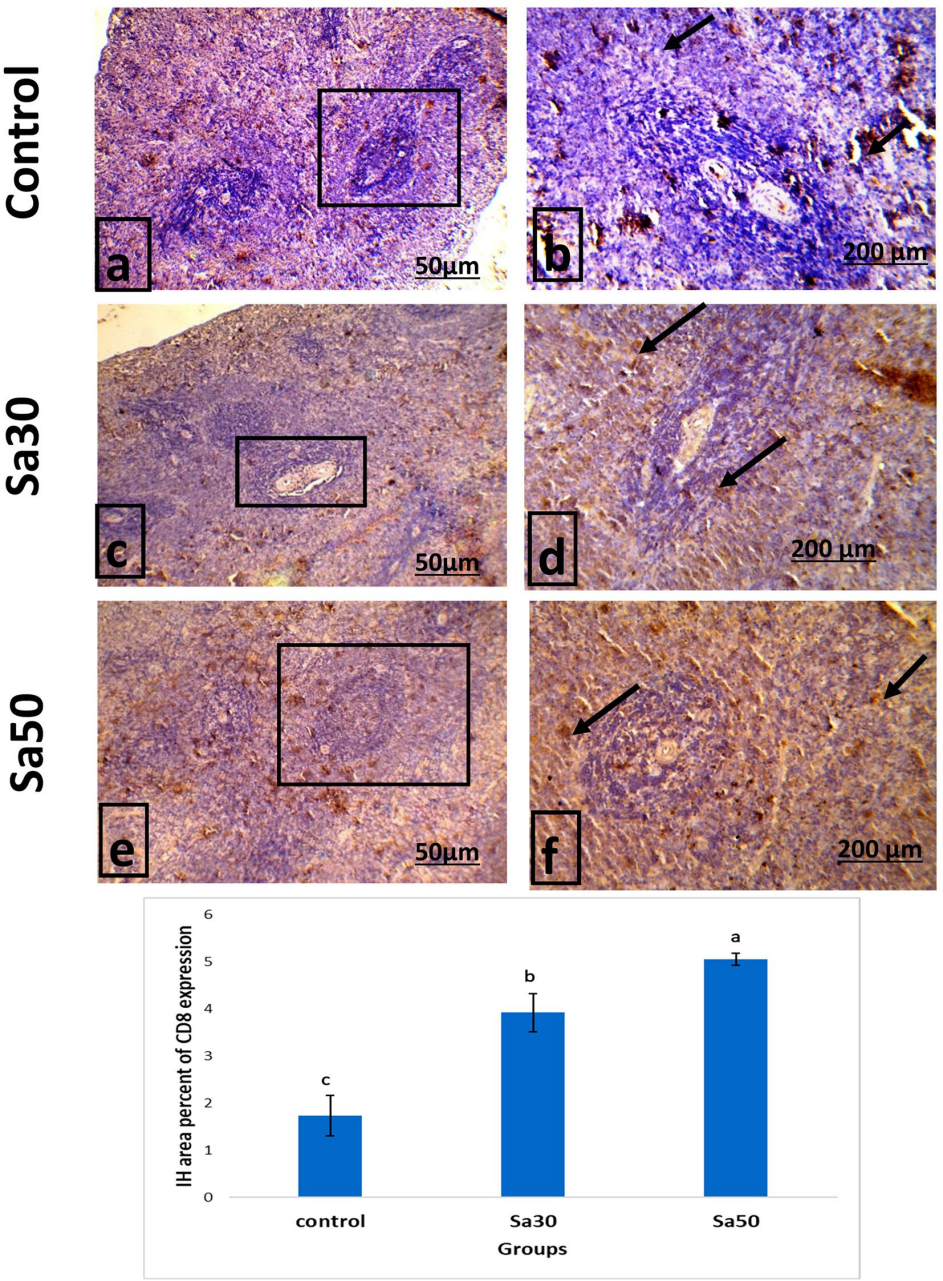
Immunohistochemistry analysis of stained sections of the spleens of control and the groups fed *nigella sativa* seed (30g/kg and 50 g/kg) showing the percent area of splenocytes showing positive CD8 expression (×100 & ×200) and a histogram showing the mean percentage of positive CD8 reactivity. Different superscript letters indicate significant differences at *P* < 0.05.

CD8 protein expression appeared brownish in splenocytes. The spleen of the control group showed very weak expression of CD8, whereas the spleens of the Sa30 and Sa50 groups showed higher expression than those of the control group (Fig. 2). The percent area of splenocytes showing positive immunohistochemical expression of CD8 in the Sa30 and Sa50 groups was significantly (*P* < 0.05) higher than that in the control group.

**Figure 3:**
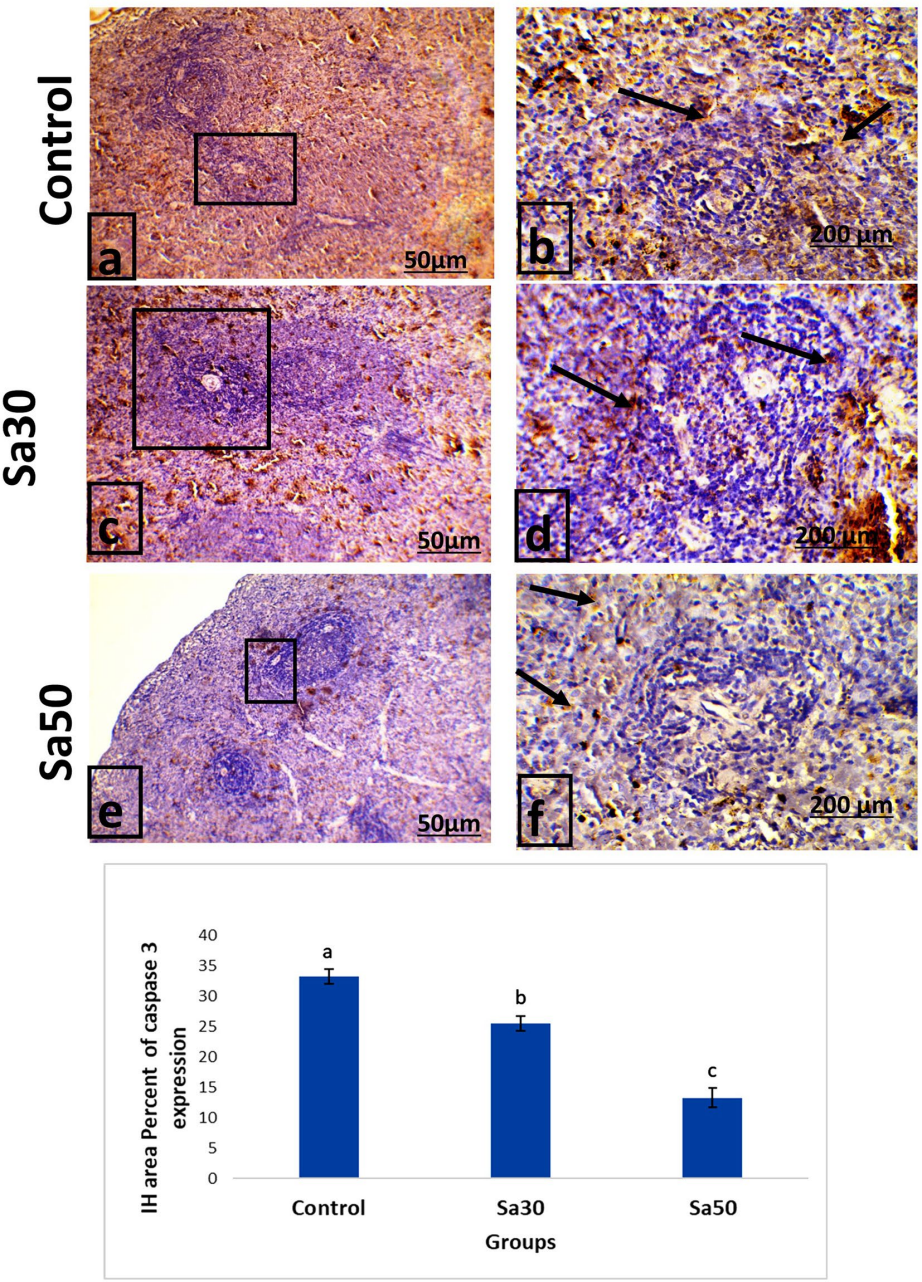
Immunohistochemistry analysis of stained sections of the spleen of rats belonging to the control group and the groups fed *Nigella sativa* seeds (30 g/kg and 50 g/kg). The percent area of splenocytes showing positive caspase-3 protein expression is shown (arrow, ×10 & ×200 magnification). The mean percentage of positive caspase-3 reactivity is shown in the histogram. Different superscript letters indicate significant differences at *P* < 0.05.

In contrast, the spleen of the control group showed strong expression of caspase-3, as demonstrated by brown coloration. In contrast, the spleens of the Sa30 and Sa50 groups showed a greater decrease in caspase-3 protein expression compared with the control group (Fig. 3). The percent area of splenocytes showing positive immunohistochemical expression of caspase-3 protein was significantly (*P* < 0.05) decreased in rats fed 30 and 50 g/kg *Nigella sativa* seeds daily for 30 days compared with those in the control groups.

**Figure 4:**
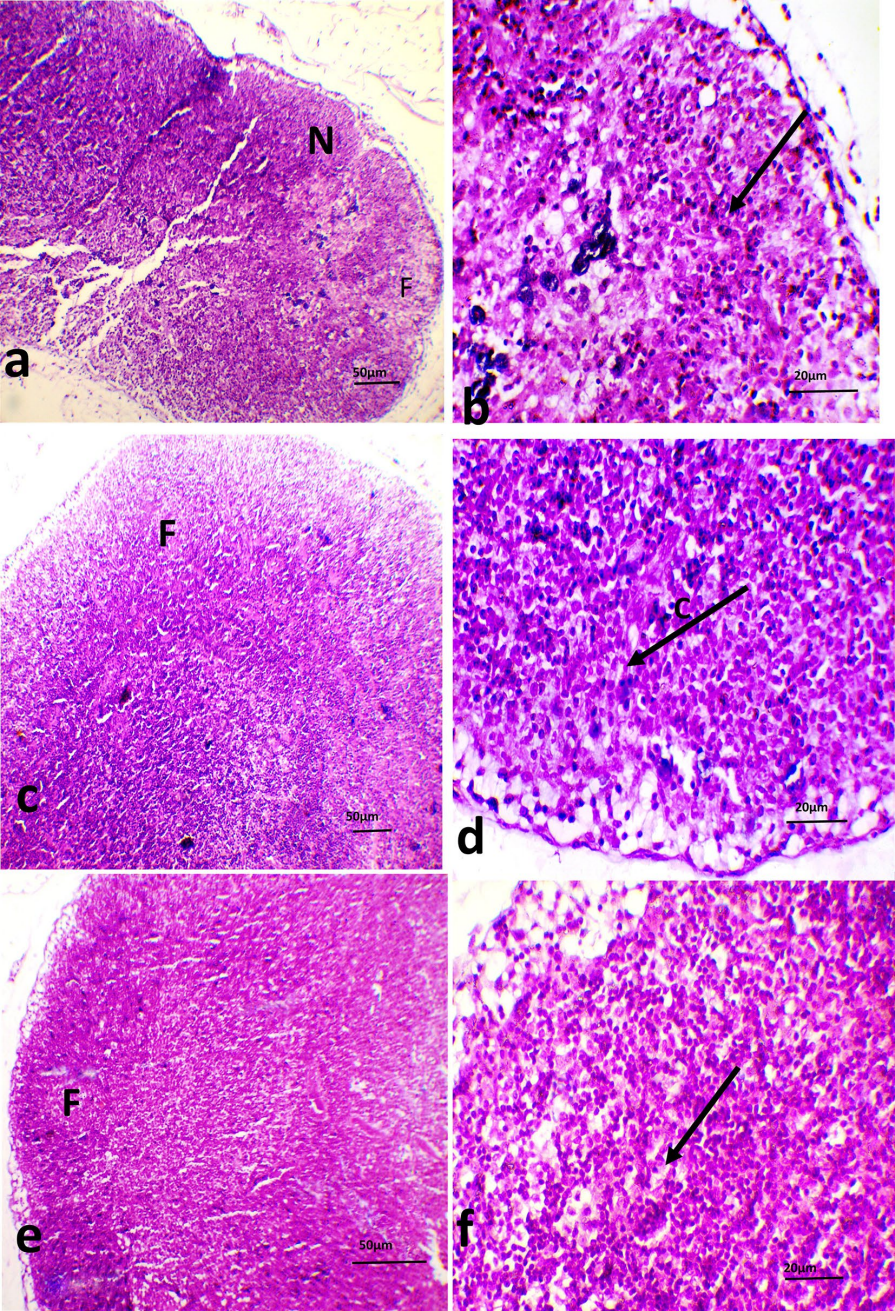
Photomicrograph of popliteal lymph nodes. (**a** & **b**) The popliteal lymph nodes from a control rat show severe degeneration with a mild necrotic area (N) and a slight increase in the number of lymphocytes (arrow). (**c** & **d**) The popliteal lymph nodes from a rat belonging to the Sa30 group showed lymphoid follicles (**f**) with germinal centers and a slight increase in the number of lymphocytes (arrow). (**e** & **f**) The popliteal lymph nodes of a rat belonging to the Sa50 group showed parafollicular hyperplasia (**f**) and a slight increase in the number of lymphocytes (arrow) (H&E stain, ×5 and ×10 magnification).

The popliteal lymph nodes of the control group injected with PHA (Fig. 4a & d) showed a reduction in the cortex width. The lymphoid follicle with a pale staining area (germinal center) was poorly demarcated. Reactive inflammatory hyperplasia of the lymph node showed a slight increase in the number of lymphocytes extending to the medulla and dispersed throughout the whole section. Severe degeneration with a mild necrotic area and lymphatic sinus ectasia were also observed. The histological inspection of the popliteal lymph nodes of the Sa30 group (Fig. 4b & e) and Sa50 group (Fig. 4c & f) showed medullary lymphoid hyperplasia with a slight increase in the number of lymphocytes that extended to the medulla and distributed throughout the whole section. The lymph nodes of the rats belonging to the Sa50 group showed parafollicular hyperplasia.

**Figure 5:**
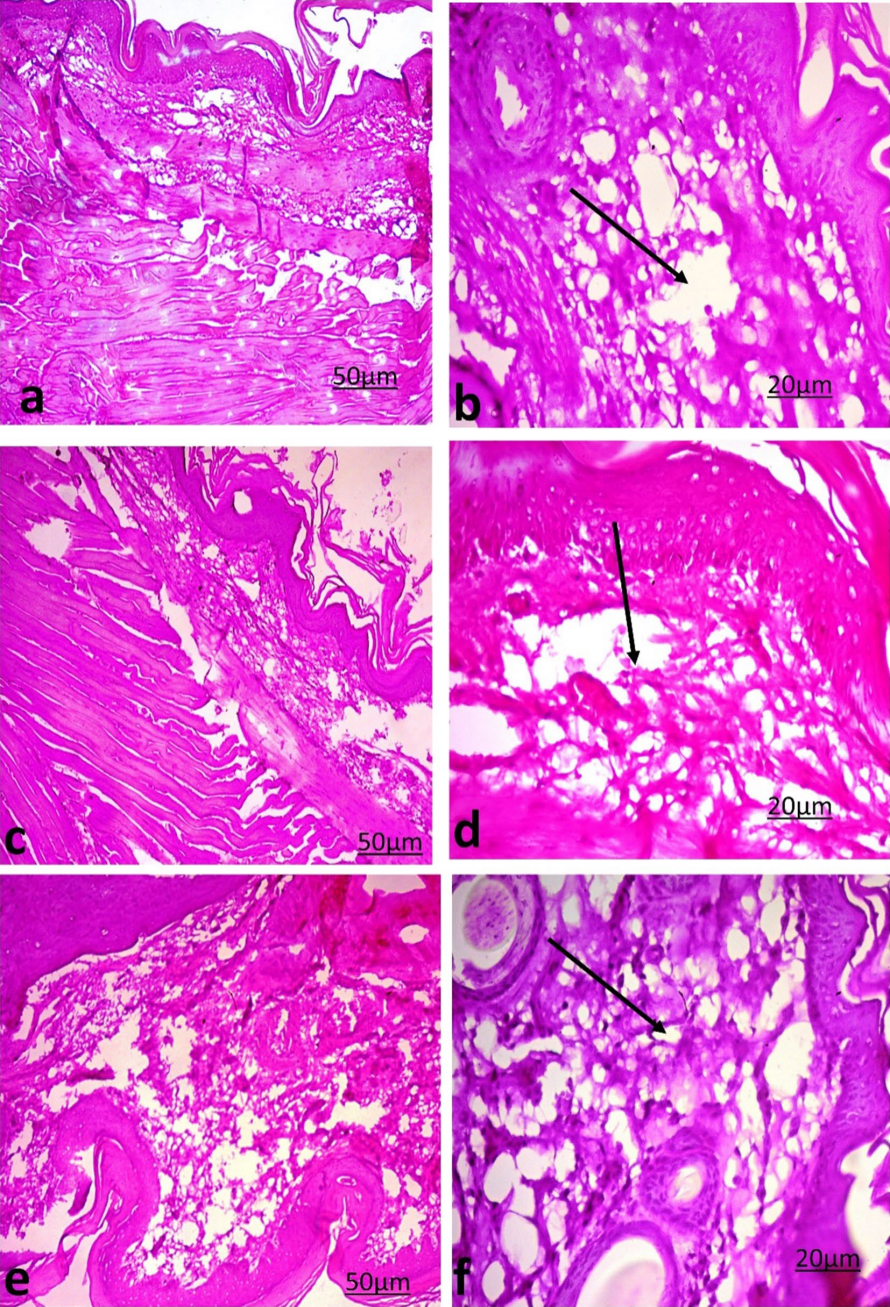
Representative photographs from footpad biopsies of PHA-injected model animals showing the effect of *Nigella sativa* seeds against PHA-induced inflammation in rats. Control rats (**a** & **b**), rats fed 30 mg of *Nigella sativa* seeds in the diet for 30 days (**b** & **c**), rats fed 50 mg of *Nigella sativa* seeds in the diet for 30 days (**c** & **d**). → : lymphocytic infiltration and edema in the dermis (H&E stain, ×100 and ×200 magnification).

The footpads of the rats were analyzed 24 h after PHA injection (Fig. 5). The control footpad exhibited marked cellular diffuse infiltration in the connective tissue with edema in the dermis, whereas the footpads of male rats fed *Nigella sativa* seeds showed an increased inflammatory response with higher lymphocyte infiltration and increased edema in the dermis compared with the control group.

## Discussions

*Nigella sativa* seeds are one of the most frequently used plants in traditional medicine. Crude *Nigella sativa* seeds have been widely used as a remedy for several diseases in prophetic medicine^14^. These seeds play a significant role as anti-inflammatory and antioxidant agents and immunological activators^15^. Some researchers have demonstrated that *Nigella sativa* influences the immune system; specifically, this species can increase the antibody response^16^ and ameliorate inflammation and immunological attacks^17^. Therefore, the current study investigated the cellular-mediated immunomodulatory action of *Nigella sativa* seeds on male adult Wister rats and their influence on performance. To the best of our knowledge, the current study provides the first experimental evidence showing that dietary *Nigella sativa* has immunomodulatory properties against 10% PHA in rats. Additionally, no previous studies have described the immune response of rats fed *Nigella sativa* seeds to the PHA skin test. The phenolic, flavonoid and antioxidant components of plants show ecological differences among cultivated plants^18^. Analyses of *Nigella sativa* seeds have revealed strong antioxidant free radical DPPH scavenging activity, which may be attributed to the high levels of phenolic and flavonoid constituents*.* Similarly, Adetuyi and Ibrahim^19^ found DPPH functions associated with phenolic and flavonoid components.

The *Nigella sativa* seed-fed groups showed significant improvements in the final body weight and FCR relative to the control group. *Nigella sativa* seeds greatly improved growth performance, and this growth performance promotion could be due to the nutritional value of key *Nigella sativa* components that contain high fatty acid percentages and essential amino acids^20^. Moreover, *Nigella sativa* exerts an enhancing effect on digestive enzymes^21^ and gastrointestinal motility^22^ and thus improves feed utilization and FCR. The previous results were in agreement with those reported by Ahmed and El-Sayed^8^.

The spleen represents an enlarged lymphatic tissue responsible for the clearance of damaged old particles from the body and foreign particles from the blood^23^. In the present study, dietary *Nigella sativa* seeds at a dose of 50 g/kg were found to increase the relative splenic weight. Moreover, the histomorphometry of the spleens from both the Sa30 and Sa50 groups showed significant improvement compared with that of the control spleens. These results were consistent with those reported by Ghonime et al.^24^, who found that *Nigella sativa* exerts a lymph-regenerating effect in lymphoid organs^25^. This finding illustrates the role of *Nigella sativa* in increasing the splenic weight.

According to our results, feeding *Nigella sativa* seeds resulted in a significant increase in catalase activity and TAC rates compared with those found with the control group. These results suggested that feeding *Nigella sativa* seeds contributes to cellular protection by providing a source of antioxidant molecules and indirectly stimulating the activity of these enzymes^26^. The active ingredients of *Nigella sativa* seeds include p-hydroxybenzoic acid, chlorogenic acid, catechin, and sinapic acid. Ferulic acid, p-coumaric acid and kaempferol has been shown to have antioxidant effects and ROS scavenging potential, which results in the promotion of higher antioxidant enzyme levels. The abolishment of oxidative stress is closely associated with the promotion of body weight gain^27^. Moreover, the antioxidant potency of *Nigella sativa* seed ingredients could be attributed to their immunostimulant effect^13,28^, as demonstrated by increased TNF-α and IF-γ levels in response to PHA stimulation and by higher splenic lymphoproliferation due to increased histomorphometric parameters in the *Nigella sativa*-treated groups.

Phytohaemagglutinin (PHA) is a mitogen-derived plant that provokes leucocyte recruitment in both innate and adaptive immune responses at the place of inoculation, resulting in quantifiable tissue swelling that could induce such an immune response^29^. The current results demonstrated a significant increase in the intumesce index in the Sa50 group. The intumesce index is expressed as the difference corresponding to the swelling response and is regarded as an index of immune responsiveness^29^. These results were parallel to the observed increases in cellular infiltration, edema and lymphocyte infiltration in the PHA-injected paws of the Sa30 and Sa50 groups. The increases in IF-γ, IL-12 and TNF-α, as cellular immune-promoting cytokines, observed in the Sa30 and Sa50 groups confirmed the immunostimulatory effect of *Nigella sativa*.

Interleukin 12 (IL-12) is an important immunomodulatory cytokine that is manufactured by macrophages, dendritic cells and antigen-presenting cells. The production of this cytokine during infection adjusts the innate immune responses and determines the adaptive immune response sequence to be elicited. Additionally, IL-12 can evoke the assembly of IF-γ from T helper type 1 (Th1), activated CD8 + T cells and natural killer cells, which, in turn, aggravate macrophages to destroy intracellular organisms^30^. IF-γ prompts the differentiation of CD4 + T cells into Th1 cells that further produce IF-γ^31^. Moreover, IL-12 induces the production of TNF-α, which plays a pivotal role in immune regulation by monitoring lymphocyte proliferation, survival and apoptosis via paracrine/autocrine signals^32^. The latter function of TNF-α is concerned with the maintenance of immune homeostasis and self-tolerance^33^. The crosstalk between the innate and adaptive immune systems that is arbitrated by IL-12 and IF-γ plays a substantial role in infectious agent control. The present study revealed significant promotion of the levels of IF-γ, IL-12 and TNF-α in the Sa30 and Sa50 groups, and this effect appeared to be dose-dependent. The present data were in agreement with those reported by Gholamnezhad et al.^34^. These results explained the cell-mediated immunostimulatory effect of *Nigella sativa*: IL-12 enhanced IF-γ, which acted in an autocrine and paracrine manner to increase CD8 + cell activity. Both γ-IF and TNF-α can stimulate macrophage activity to eliminate infectious agents^35,36^.

*Nigella sativa* seed-treated rats showed a significant increase in CD8 immunoreactivity in splenocytes. Salem et al.^37^ reported similar results; these researchers found that the addition of thymoquinone stimulated CD8 + T cells and markedly increased IF-γ production. Our findings indicate that the expression of CD8 may be linked to the activation state of T cells injected with PHA. CD8 + cells are a crucial constituent of the cellular immune response, and their frequency is increased during conditions in which the immune system is activated by infection^38^, autoimmune disease or transplantation^39^. CD8 + T cells produce cytokines such as IF-γ and TNF-*α*, and the latter is a proinflammatory cytokine that initiates apoptotic gesturing and inhibits viral replication as well as gene expression^40^. Additionally, CD8 + T cells could directly attack and induce cytolysis to infected targets^41^.

The *Nigella sativa* seed-treated rats showed a significant dose-dependent decrease in the expression of caspase-3 splenic immunoreactivity. Similarly, Salem^6^ found that the spleen of mice treated with *Nigella sativa* exhibited decreased apoptosis rates. Numerous lines of evidence have indicated that *Nigella sativa* seeds can modulate proinflammatory cytokines as multiple cell signaling molecules^42^, apoptotic proteins^43^ and antioxidants^6^. The reduction in caspase-3 protein expression observed in the groups administered *Nigella sativa* seeds indicated the antiapoptotic potential of these seeds, which could be attributed to their antioxidant ingredients. This effect was demonstrated by elevated TAC and catalase activities in the *Nigella sativa-*treated groups*.* Moreover, the reduction of splenic caspase-3 denoted an active dynamic status in this organ in response to PHA injection that was augmented by increased splenic CD8 expression as well as higher serum IF-γ, IL-12 and TNF-α. This scenario was reflected in the increases in edema and inflammatory cell infiltration observed in the PHA-stimulated foot pads as well as the higher intumesce index. Moreover, the areas of white pulp, periarterial lymphoid sheath and the germinal center were significantly increased in the Sa30 and Sa50 groups, reflecting an active splenic performance with lower apoptosis.

Histological assessment of lymph nodes is crucial for comprehending the immunologic effects of chemicals^44^. PHA stimulates a characteristic pattern of reaction in lymph nodes. The control group injected with PHA showed a reduced lymphoid follicle size with medullary lymphoid hyperplasia, severe degeneration with a mild necrotic area and lymphatic sinus ectasia. The histopathological alteration of the lymph node is due to its inflammatory response to PHA injection, which triggers an immunologic response, as described by O'Dowd et al.^45^. The lymph nodes from the Sa30 and Sa50 groups showed medullary lymphoid hyperplasia, a reduced lymphoid follicle size and less notable distortion of the architecture compared with the control lymph nodes. Hyperplasia occurring in the lymph node is an acute immune response to antigens^46^. Therefore, PHA acts as an antigen stimulator. The lymph nodes of the Sa50 group showed parafollicular hyperplasia in which the follicle was pushed toward the periphery of the node beneath the capsule, which may be a response to reactive hyperplasia of the lymph node^47^. The *Nigella sativa* immunologic response shows an explicit dose-dependent trend, and the higher dose tended to result in the least damage and higher lymphocyte infiltration in the popliteal lymph node tissues of the experimental rats.

Based on all the previous data, it appears that *Nigella sativa* induces a favorable cell-mediated immune response to PHA injection through its antioxidant active ingredients that positively influence the weight gain and FCR. The effect of *Nigella sativa* seeds was represented by an increased IL-12 level, which promoted CD8 and IF-γ production, and an increased TNF-α level, which could effectively respond to infectious agents. This active immunomodulatory cell-mediated immune response was accompanied by an active splenic state consisting of increased CD8 expression and a reduced level of caspase-3, which serves as an apoptotic marker. These findings were manifested by an improved lymphoid histomorphometry in the spleen with increased chemotaxis and inflammatory reactions at the site of PHA injection. Additionally, the popliteal lymph nodes of the PHA-injected legs showed lymphoid hyperplasia.

## Conclusion

The current study established that *Nigella sativa* seeds can potentially be introduced into foods because these seeds exerted a positive effect by boosting overall growth output parameters, the FCR and the immunological response. The later effects of these seeds are due to their antioxidant constituents, which induce cell-mediated cytokine production, promote splenic CD8 T cells and reduce splenic caspase-3 expression. Based on this study, *Nigella sativa* seeds could be useful as a dietary supplement that exerts a positive modulatory effect on the cell-mediated immune response to disease. Thus, *Nigella sativa* dietary supplementation could be beneficial in viral or bacterial infections in which the cellular-mediated immune response plays a pivotal role.

## Methods

### Plant material

Black seeds (100% organic *Nigella sativa* seeds), produced in the Oromia region of Ethiopia, were purchased from a local market (Commercial Registration No 228/056/572) in 2019. The seeds material complies with international guideline and regulation. The seeds were morphologically identified by Botany Department, Faculty of Science, Suez Canal University, Egypt, (voucher number: 54782) and kept within the Prophetic Medicine Foundation, Ismailia, Egypt. The whole seeds were shriveled daily in a blender and mingled well with a basal diet just before its administration to the rats. A quantitative phytochemical constituent analysis of *Nigella sativa* seeds was performed using protocols previously described by Silahtaroglu et al.^48^.

### Phytochemical analysis of *Nigella sativa* seeds and antioxidant activity

The shriveled *Nigella sativa* seeds were subjected to hydrolysis by vigorous shaking with 2 M NaoH for 4 h then PH was adjusted to 2 via adding 6 M hydrochloric acid. Centrifugation was performed to the later contents for 10 min at 5000*g* and the supernatant was harvested. Extraction of phenolic compounds was performed by 50 mL ethyl ether and ethyl acetate (1:1). Separation of the organic phase was done with further evaporation of the solvent at 45 °C and the residues were re-dissolved in 2 mL methanol. A phytochemical screening of phenolic compounds in the residue was performed via Agilent Technologies 1100 series liquid chromatograph according to Oladeji et al.^49^ The injection volume was 50 µL with solvent A was acetonitrile and solvent B was 2% acetic acid in water (v/v). The analytical column was Eclipse XDB-C18 (Phenomenex, Torrance, CA). The flow rate was adjusted at 0.8 mL/min for 60 min and the gradient programme was as follows: 100% B to 85% B in 30 min, 85% B to 50% B in 20 min, 50% B to 0% B in 5 min and 0% B to 100% B in 5 min. Peaks were identified by congruent retention times and UV spectra and compared with those of the standards.

The total phenolic content in *Nigella sativa* seeds was estimated according to Khattak et al*.*^50^. 2 mL of 2% aqueous NaCO_3_ solution were mixed with 100 µL of the black seeds extract then 100 µL of 50% Folin–Ciocalteu reagent were supplemented to the mixture. Thorough shaking was done and the tube was set aside for 1 h. Green–blue complex was formed that measured via spectrophotometer (Pharmacia, Uppsala, Sweden), against a blank control at 750 nm. The total phenolic contents were deliberated on the basis of a calibration curve of gallic acid.

The total flavonoid content in *Nigella sativa* seeds extract was estimated based on the method described by Zilic et al*.*^51^. One hundred microliters of the black seeds extract were mixed with 300 µL of 5% sodium nitrite (NaNO2) then 300 µL of a 10% AlCl_3_ solution were added 6 min later. The volume of the later mixture was adjusted to 2.5 mL using distilled water. 7 min later, 1.5 mL of 1 M NaOH was added, and the mixture was centrifuged at 5000*g* for 10 min. The supernatant was harvested for measuring absorbance was at 510 nm against the solvent blank. The total flavonoid content was deliberated on the basis of a calibration curve of catachin.

The antioxidant activity of the seeds was assessed using the method described by Mariod et al.^52^. A total of 100 µL of the black seeds extract were added to 0.5 mL of a 0.05% methanolic solution of 1,1-diphenyl-2-picrylhydrazyl (DPPH) and incubated in dark condition at 23 °C for 30 min. The absorbance was recorded at 515 nm using a spectrophotometer (Pharmacia, Uppsala, Sweden). The absorbance of control DPPH radical, i.e. without black seed extract, was recorded. The data is commonly reported as inhibition concentration 50 (IC_50)_ that provides 50% of DPPH inhibition in definite time. All measurements were done in triplicate.

### Experimental animals

Eighteen young adult male Wistar rats (weighing 105–115 g and aged 3 months). At this age, the rats were at a sexually mature stage equivalent to the beginning of human puberty^53^. The rats were obtained from the Animal House in Suez Canal University, Faculty of Sciences, Ismailia, Egypt. The rats were acclimated for 2 weeks in clean cages under standard conditions and had free access to water and feed. The animals’ room was subjected to a natural daylight rhythm and had adequate ventilation throughout the experimental period (30 days).

### Experimental design and diets

The experimental rats were randomly divided into three groups (6 rats in each group): the control group and two treatment groups. The rats of the control (untreated) group were fed a basal formulated diet that provided the nutritional requirements of rats according to the Nutrient Requirements of Laboratory Animals^54^. The rats in the two treatment groups (Sa30 and Sa50 groups) were fed the basal diet supplemented with 30 g/kg and 50 g/kg black seed powder, respectively, for 30 days. The 30-day treatment period was selected according to Chakrabarti et al.^55^, who demonstrated that this period was the best duration for inducing an immune response by black seed supplementation.

### Growth performance parameters

The feed intake and weights of each experimental animal/group were recorded at the beginning and end of the experiment to monitor various growth output parameters using the following formulae:

$$\begin{gathered} {\text{Weight gain }}\left( {{\text{g rat}}^{{{-}{1}}} } \right) = {\text{W}}_{{\text{f}}} - {\text{W}}_{0} {.} \hfill \\ {\text{Feed conversion ratio }}\left( {{\text{FCR}}} \right) \, = {\text{FI}}/\left( {{\text{W}}_{{\text{f}}} - {\text{W}}_{0} } \right). \hfill \\ \end{gathered}$$

where W_0_ and W_f_ are the initial and final weights of the rats in each group, respectively, and FI is the feed intake.

### Phytohemagglutinin injection and intumesce index

The left foot of each experimental rat/group was injected with 0.1 mL of 10% (v/v) phytohemagglutinin (PHA) (L 9017, Sigma Aldrich, USA) in PBS. The right footpad of the same rat was used as a control by inoculation with 0.1 mL of PBS^56^. The rats were maintained for 24 h, and the thickness of the lateral and dorsoventral aspects of the left footpad at the injection point was then assessed with a manual micrometer. The same person made the injections and steps to reduce errors. The intumesce index was determined as the (measured ankle size-primary ankle size)/primary ankle size.

### Blood and tissue collection

The rats were anesthetized with tetrahydrofuran inhalation anesthesia after overnight fasting. While the rats were under the effect of the anesthesia, blood samples were drawn at the end of the experimental period from the orbital venous plexus into plain tubes. Sera were isolated and maintained at − 70 °C until further analyses of antioxidants (catalase and TAC), IF-γ, IL-12 and TNF-α.

The rats were sacrificed, and the spleen was excised, microscopically examined and weighed to calculate the weight relative to the body weight. Additionally, the popliteal lymph nodes from the PHA- and PBS-injected paws were excised. Both the spleen and popliteal lymph nodes were fixed in 10% neutral formalin solution for histopathological examination and immunohistochemical detection.

### Catalase activity and TAC

The catalase activity and TAC in the sera were measured using a colorimetric method and kits (K773 and K274; BioVision Inc., Milpitas, CA, USA) according to the manufacturer’s instructions.

### IF-γ, IL-12 and TNF-α levels

The IF-γ, IL-12 and TNF-α levels were assayed using a rat enzyme-linked immunosorbent sandwich assay (ELISA) kits (Thermo Fisher Scientific, USA) according to the manufacturer’s instructions: the IF-γ ELISA kit (BMS629) has a detection limit of 11.0 pg/mL; the serum IL-12 ELISA kit (KRC0121) has a detection limit of < 2.5 pg/mL; and the TNF-α ELISA kit (BMS621) has a detection limit of 9.9 pg/mL. The kits were carefully checked to assess their sensitivity, specificity and reliability. A spectrophotometer and a microplate reader (Biotech, USA) were used to measure the absorbance.

### Histopathology and immunohistochemistry examination

Formalin-fixed spleen and foot paws were progressively dehydrated, cleared, and then submerged in paraffin wax. Numerous 5-μm sections were obtained and stained with hematoxylin and eosin (H&E) according to Bancroft and Gamble^57^ for histopathological examination. Histomorphometric analysis to spleen was performed. From 6 experimental rats in each group, 3 slides of each organ were obtained and examined. Immunohistochemistry of splenic caspase-3 and CD8 was performed using primary antibodies against caspase-3 (#PAI29157, Thermo Scientific Co., USA) and CD8 (Cat. No. 6A242, Santa Cruz, CA, USA) at dilution rates of 1:1000 and 1:200, respectively. The procedures were performed with secondary polyvalent biotinylated antibodies according to the methodology described by Zhao et al.^58^ and Elgawish and Abdelrazek^59^. For histomorphometric and immunohistochemical (IHC area %) analyses of spleen, seven random images of each slide were analyzed by ImageJ software. The tissues were examined by experiments blinded to the group to which the samples belonged.

### Statistical analysis

The results are presented as the means ± standard errors of the mean (SEMs). The differences between groups were assessed by one-way analysis of variance (ANOVA) followed by Duncan post hoc multiple comparison tests (SPSS software, version 16.0; SPSS Inc., Chicago, IL, USA), and *P* < 0.05 indicated significance.

### Ethical approval

This animal experiment was performed following EU Directive 2010/63/EU and compliance with ARRIVE guideline, which was strictly followed to minimize the suffering of the animal during the experiments. The experimental work was approved by the Institutional Animal Ethics Committee of Faculty of Veterinary Medicine Animal Ethics Committee, Suez Canal University, with Number: 2020041.

## Supplementary Information

Supplementary Information

## Data Availability

Data supporting findings are presented within the manuscript.
