# Supplementary material for: The effect of dietary supplementation with Nigella sativa (black seeds) mediates immunological function in male Wistar rats
Source: Sci Rep. 2021 Apr 6;11:7542. doi: 10.1038/s41598-021-86721-1 (PMC8024296; doi:10.1038/s41598-021-86721-1)
Supplement: Supplementary file 1 — Supplementary Information [file 41598_2021_86721_MOESM1_ESM.pdf]

Supplementary Material :

The effect of dietary supplementation with *Nigella sativa* (black seeds) mediates immunological function in male Wistar rats

Hany Salah. Mahmoud[1], Amani A. Almallah[2], Heba Nageh. Gad EL-Hak[3], Tahany Saleh. Aldayel[4]*, Heba M.A. Abdelrazek[5], Howayda E. Khaled[6]

[1]Center of Scientific Foundation for Experimental Studies and Research, Ismailia, Egypt, 41511.

[2]Anatomy and Embryology Department, Faculty of Medicine, Suez Canal University, Egypt, 41522.

[3]Zoology Department, Faculty of Sciences, Suez Canal University, Egypt, 41522.

[4]Nutrition and Food Science, Department of Physical Sport Sciences, Princess Nourah bint Abdulrahman University, Riyadh, Saudi Arabia, 11671.

[5]Department of Physiology, Faculty of Veterinary Medicine, Suez Canal University, Ismailia, Egypt, 41522.

[6]Zoology Department, Faculty of Sciences, Suez University, Egypt, 43533.

***Corresponding author**

Tahany Saleh Aldayel, Nutrition and Food Science, Department of Physical Sport Sciences, Princess Nourah bint Abdulrahman University, Riyadh, Saudi Arabia, 11671. TSALdayel@pnu.edu.sa

1g in 2ml

===

```
Injection Date  : 3/8/2020 5:37:39 PM
Sample Name     : black seed                    Location : Vial 34
Acq. Operator   : A
Acq. Instrument : Instrument 1                Inj Volume : 10 µl
Acq. Method     : C:\HPCHEM\1\METHODS\PHENOLS2.M
Last changed    : 3/8/2020 5:36:13 PM by A
                  (modified after loading)
Analysis Method : C:\HPCHEM\1\METHODS\PHENOLS2.M
Last changed    : 3/8/2020 6:44:33 PM by A
                  (modified after loading)
```

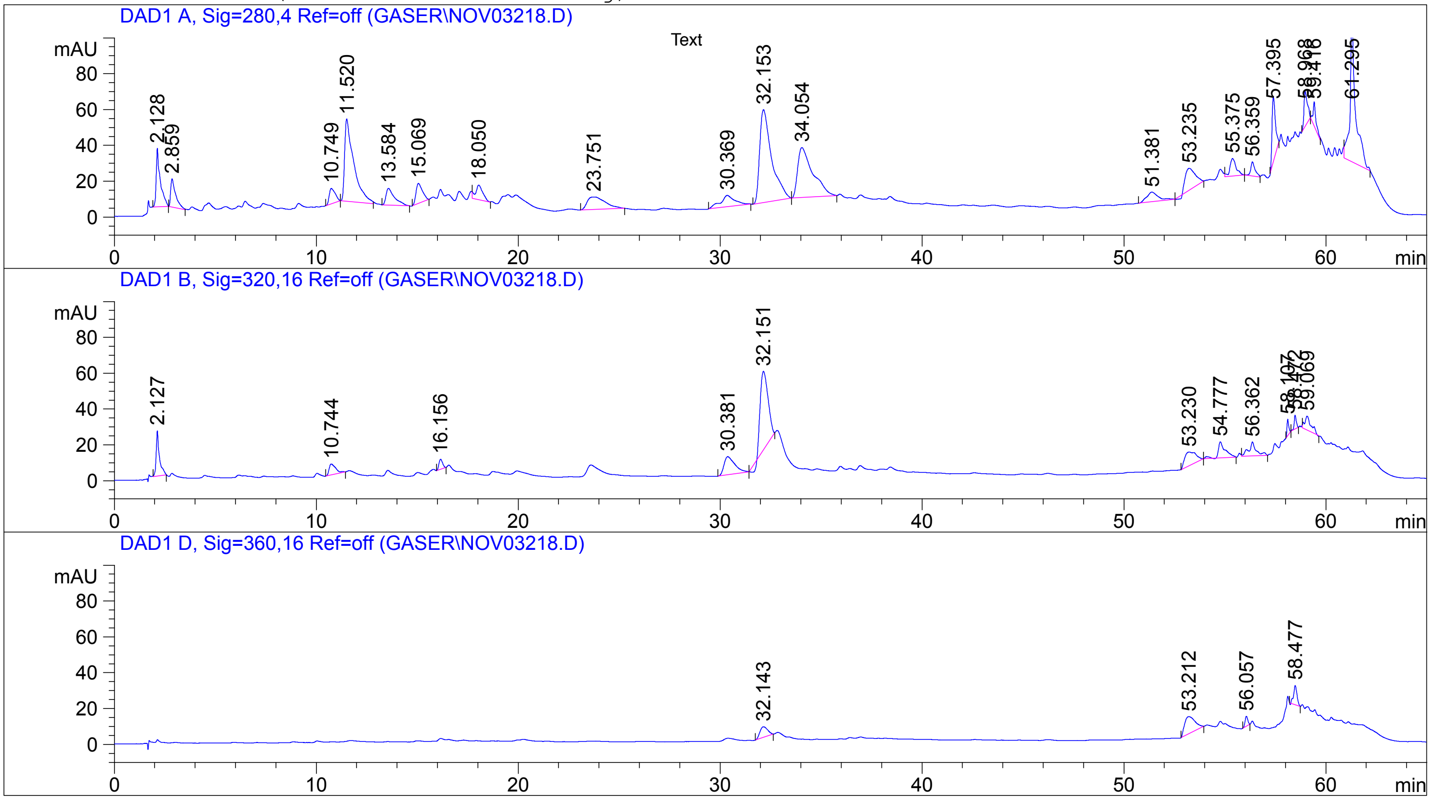

===

Area Percent Report with Performance and Noise

===

```
Multiplier              :       1.0000
Dilution                :       1.0000
Use Multiplier & Dilution Factor with ISTDs
```

Signal 1: DAD1 A, Sig=280,4 Ref=off
Results obtained with enhanced integrator!

RetTime [min]	k'	Area [mAU*s]	Height [mAU]	Symm.	Width [min]	Plates	Resol ution	Signal /Noise
2.128	-	459.50623	32.56649	0.37	0.1889	703	-	-
2.859	-	269.06653	15.78131	0.33	0.2311	848	2.05	-
10.749	-	175.76259	8.50560	0.53	0.3733	4593	15.34	-
11.520	-	1446.98926	45.37874	0.29	0.4500	3631	1.10	-
13.584	-	277.69363	9.39817	0.36	0.4033	6284	2.84	-
15.069	-	247.44295	11.04121	0.57	0.3900	8271	2.20	-
18.050	-	203.38579	8.20631	0.67	0.5191	6697	3.85	-
23.751	-	433.84018	6.92487	0.46	1.0200	3004	4.35	-
30.369	-	313.22372	6.16251	0.74	0.6933	10629	4.54	-
32.153	-	2125.76782	51.62777	0.39	0.5867	16641	1.64	-
34.054	-	1445.71387	27.71048	0.32	0.7267	12167	1.70	-
51.381	-	217.88692	5.34745	0.55	0.5933	41545	15.42	-
53.235	-	404.67261	12.17032	0.75	0.7700	26480	1.60	-

RetTime [min]	k'	Area [mAU*s]	Height [mAU]	Symm.	Width [min]	Plates	Resolution	Signal /Noise
55.375	-	211.65732	9.79341	0.64	0.2978	191581	2.35	-
56.359	-	119.11971	7.85561	0.98	0.2000	439929	2.32	-
57.395	-	429.21899	36.64047	0.51	0.2022	446269	3.02	-
58.968	-	234.59596	19.77241	0.57	0.2453	320062	4.13	-
59.416	-	130.45879	13.37119	0.64	0.1093	1636126	1.48	-
61.295	-	1530.53687	81.02058	0.49	0.1767	666888	7.72	-

Signal 2: DAD1 B, Sig=320,16 Ref=off
Results obtained with enhanced integrator!

RetTime [min]	k'	Area [mAU*s]	Height [mAU]	Symm.	Width [min]	Plates	Resolution	Signal /Noise
2.127	-	285.19550	25.31251	0.48	0.1383	1310	-	-
10.744	-	139.19051	6.13427	0.36	0.3833	4352	19.41	-
16.156	-	69.88361	5.59309	0.71	0.2089	33141	10.74	-
30.381	-	374.96072	10.04964	0.42	0.6100	13742	20.41	-
32.151	-	1133.62219	43.60803	0.69	0.4833	24513	1.90	-
53.230	-	291.10495	7.60167	0.57	0.9467	17516	17.32	-
54.777	-	220.88582	9.00736	0.94	0.2867	202277	1.47	-
56.362	-	200.68759	7.68703	0.74	0.2133	386696	3.73	-
58.107	-	55.57148	9.11353	0.59	0.0990	1906661	6.56	-
58.472	-	55.76585	7.73669	1.15	0.1120	1509956	2.03	-
59.069	-	169.01936	7.86634	0.45	0.3400	167213	1.55	-

Signal 3: DAD1 D, Sig=360,16 Ref=off
Results obtained with enhanced integrator!

RetTime [min]	k'	Area [mAU*s]	Height [mAU]	Symm.	Width [min]	Plates	Resolution	Signal /Noise
32.143	-	159.10129	5.97260	0.69	0.4833	24501	-	-
53.212	-	341.44943	9.55651	0.53	0.7600	27158	19.91	-
56.057	-	51.61699	5.56294	0.89	0.1517	756820	3.67	-
58.477	-	125.40991	10.68334	1.07	0.1689	664171	8.87	-

==

*** End of Report ***
